# Increased implant thickness in mechanically aligned off‐the‐shelf total knee replacement compared to bone resection

**DOI:** 10.1002/jeo2.70307

**Published:** 2025-06-12

**Authors:** Filippo Calanna, Riccardo Compagnoni, Luca Tanel, Alessandra Menon, Alessio Maione, Carlo Minoli, Paolo Ferrua, Pietro Simone Randelli

**Affiliations:** ^1^ 1° Clinica Ortopedica, ASST Centro Specialistico Ortopedico Traumatologico Gaetano Pini‐CTO Milan Italy; ^2^ Department of Biomedical, Surgical and Dental Sciences Università degli Studi di Milano Milan Italy; ^3^ Università degli Studi di Milano Milano Italy; ^4^ Department of Biomedical Sciences for Health, Laboratory of Applied Biomechanics Università degli Studi di Milano Milan Italy; ^5^ U.O.C Week Surgery, ASST Centro Specialistico Ortopedico Traumatologico Gaetano Pini‐CTO Milan Italy; ^6^ Department of Biomedical Sciences for Health, Research Center for Adult and Pediatric Rheumatic Diseases (RECAP‐RD) Università degli Studi di Milano Milan Italy

**Keywords:** bone–implant mismatch, knee, mechanical alignment, off‐the‐shelf knee prothesis, total knee arthroplasty

## Abstract

**Purpose:**

Total knee arthroplasty (TKA) with mechanical alignment (MA) aims to align the leg neutrally, optimising stability and load distribution. To achieve this, bone cuts are performed, modifying the constitutional alignment of the knee and determining a mismatch between the implant thickness and the bone resection across the various compartments of the knee (bone–implant mismatch). This study aims to quantify the bone–implant mismatch in TKA with MA using an off‐the‐shelf implant and investigate how this ratio is influenced by the preoperative coronal knee alignment phenotype.

**Methods:**

Data from 100 patients who underwent primary off‐the‐shelf TKA with MA with a medial pivot design implant from January 2021 to September 2023 were analysed. Preoperative alignment phenotype was determined via long‐leg weightbearing radiographs. During surgery, bone resections were measured with a caliper for each compartment. Bone–implant mismatch was determined for each compartment, analysing differences between medial and lateral compartments and the influence of preoperative alignment phenotype.

**Results:**

The overall implant thickness was 14.2% greater than the bone resection. In the patello‐femoral compartment the implant thickness was 16.0% ± 19.0 less than the bone resection. Bone‐implant mismatch was observed in the tibio‐femoral joint across all the compartments with a significant difference between medial and lateral (*p* < 0.001). No significant differences in bone–implant mismatch were found based on preoperative alignment phenotype.

**Conclusions:**

In mechanically aligned off‐the‐shelf TKA, implant thickness exceeds bone resection by an average of 14.2%, with a consistent bone–implant mismatch observed in all tibiofemoral compartments—significantly greater medially than laterally. Conversely, in the patellofemoral joint, bone resection surpasses implant thickness. Preoperative coronal alignment phenotype does not significantly influence this mismatch. These findings highlight a systematic discrepancy between bone resections and implant geometry, suggesting potential benefits from adjusted surgical techniques or implant design modifications to improve anatomical congruence and joint kinematics.

**Level of Evidence:**

Level III.

**Abbreviations**
AP: anteroposterior
BMI: body mass index
CPAK: coronal plane alignment of the knee
CT: computed tomography
HKA: hip‐knee‐ankle angle
IQR: interquartile range
IRB: Institutional Review Board
JL: joint line
JLO: joint line obliquity
LDFA: lateral distal femoral angle
MA: mechanical alignment
MCL: medial collateral ligament
MPTA: medial proximal tibial angle
OTS: off‐the‐shelf
PCA: posterior condylar axis
PE: polyethylene
PF: patellofemoral
SD: standard deviation
TEA: transepicondylar axis
TKA: total knee arthroplasty

## INTRODUCTION

The primary goal of total knee arthroplasty (TKA) with mechanical alignment (MA) is to restore a neutral alignment with the prothesis perpendicular to the mechanical leg axis, aiming to improve stability, load distribution and patient outcomes at long term [5, 6, 14]. To achieve this, bone resections are performed, modifying the constitutional alignment of the knee and altering its joint line obliquity (JLO) [7, 21, 25, 29]. This process determines a mismatch between the component thickness and the bone resection across the various compartments of the knee (bone–implant mismatch) [26, 27]. Various factors, such as preoperative coronal knee alignment phenotype, knee laxity pattern (especially in case of gap balancing technique) and cartilage/bone wear determine the thickness of bone cuts, thus influencing bone–implant mismatch. Even the implant design seems to play a role: a recent meta‐analysis showed a higher bone–implant mismatch when using off‐the‐shelf (OTS) implants [1, 2], while custom TKA based on computed tomography (CT) reconstructions showed the potential to reduce this issue [3, 4].

Many studies have examined alignment phenotype changes from preoperative to postoperative in TKA with MA [7, 21, 25, 29] and how this theoretically determines a bone–implant mismatch [26, 27], however, none of these studies have specifically measured quantitatively the bone–implant mismatch in the different knee compartments.

This study aims to quantify the bone–implant mismatch (expressed as the percentage of extra implant thickness relative to the bone resection) in TKA with MA using an OTS implant and analyze how this is influenced by the preoperative coronal knee alignment phenotype.

The main hypothesis of the study was that the overall implant thickness, considering all the compartments of the knee, exceeds the thickness of the bone resection.

The second hypothesis was that there is bone–implant mismatch in each compartment of the knee with a significative difference medially compared to laterally in the tibio‐femoral compartment and that this differs significantly depending on the preoperative knee alignment phenotype.

## METHODS

The study was designed based on the criteria of the Declaration of Helsinki and approved by the Institutional review board (IRB No. CTS‐P‐2024‐018‐FC).

An analysis of collected data was conducted. All patients undergoing primary TKA with MA with a medial pivot implant design (Evolution® Medial‐Pivot ‐ MicroPort, Arlington, TN) between January 2021 and September 2023 were considered for this study. All the operations were performed by a single senior knee surgeon. The following inclusion criteria were adopted: primary or post‐traumatic end‐stage tricompartmental knee osteoarthritis, co‐morbidities that do not contraindicate knee replacement, a Body Mass Index (BMI) <40 kg/m^2^.

Patients were excluded in case of inflammatory osteoarthritis, ligamentous insufficiency of the knee (JLCA > 5°), patients with active infections or altered skin conditions, patients with altered neurological conditions or neurological diseases, preoperative HKA < 160° or >200° and extra‐articular deformities suitable for knee osteotomies, revision surgery and in case of mayor ligamentous release.

### Radiographic measurements

For each patient preoperative long‐leg anteroposterior weightbearing radiograph was obtained and the HKA angle calculated. Varus knee alignment was defined as an HKA angle of 177° or less, valgus knee alignment was defined as an HKA angle of 183° or more and a neutral knee alignment was defined as an HKA angle between 177° and 183° [10, 11].

### Bone resection measurements

The thickness of the bone cuts in the femur, tibia and patella in each compartment of the knee was measured with a caliper during the operation. The total thickness of the bone resection was then calculated by adding the thickness of the blade used for the cuts (1.27 mm).

### Surgical technique

In all procedures, MA was aimed. A medial parapatellar approach was used in all surgeries, performed by the same senior surgeon. The procedure begins by assessing the extension gap, followed by the flexion gap. We start with the distal femoral cut to open up the extension space, which facilitates subsequent steps of the procedure. The extension and flexion gaps were prepared utilising manual instrumentations and spacer blocks. A delicate partial subperiosteal release of the deep MCL bundle was performed to ensure better visibility of the joint and to prevent iatrogenic traumatic ruptures during the procedure. Such release did not alter the joint stability in any way during intra‐operative dynamic tests. No other releases were necessary or carried out. Patellar resurfacing was performed only in cases of severe patellofemoral arthritis with lateralized patella, following the selective resurfacing technique (i.e., bone‐on‐bone, especially if the patellofemoral joint surface was irregular, considering that this is a PS implant) [9].

The peri‐operative approach was standardised for all patients, including spinal anaesthesia and regional analgesic block, prophylactic antibiotics (Cefazolin 2 g pre‐operation, in the absence of allergies to this class of antibiotics Vancomycin 15 mg/kg was used as an alternative), and standard dose of ev. Tranexamic Acid (1 g) to reduce post‐operative blood loss, unless contraindicated. The surgical protocol adhered to established guidelines, including the use of a pneumatic tourniquet, meticulous haemostasis, and capsule closure followed by intra‐articular injection of 1 g of tranexamic acid. Tourniquet time (from inflation to release) and total surgical time (from incision to skin closure) were recorded.

### Bone resection‐implant thickness mismatch

The bone resection‐implant thickness mismatch (bone–implant mismatch) was expressed as the percentage of extra implant thickness relative to the bone resection.

For the tibial implant thickness, we considered the tibial component + polyethylene insert and for the femoral implant thickness we considered the femoral component.

It was calculated as follows: (implant thickness − bone resection)/bone resection × 100. The following values were obtained:

– percentage of extra implant thickness in the tibia: medially and laterally;
– percentage of extra implant thickness in the distal femur: medially and laterally;
– percentage of extra implant thickness in the posterior femur: medially and laterally;
– percentage of extra implant thickness in the femoro‐patellar joint: trochlea and patella.

### Statistical analysis

Statistical analyses were performed using R statistical software (Version 4.2.3; R Foundation for Statistical Computing) and GraphPad Prism (Version 6.0; GraphPad Software Inc). The Shapiro–Wilk normality test was used to evaluate the normal distribution of the sample. Continuous variables were presented as mean ± standard deviation and median [first quartile‐third quartile]. Categorical variables were expressed in numbers of cases and frequencies; their differences were tested using the chi‐square test or Fisher's exact test.

The between‐group differences for continuous variables were evaluated with the unpaired Student t‐test or Mann–Whitney test, according to the characteristics of the data distribution. The within‐group differences from baseline to post‐operative time points for continuous variables were evaluated with the Paired t‐test or Wilcoxon matched‐pairs signed rank test. according to the characteristics of the data distribution. For all analyses. the significance level was set at *p*‐value lower than 0.05.

In line with the recommendations by Hoenig and Heisey [12], we interpreted our results based on effect estimates and confidence intervals rather than relying on post hoc power calculations.

## RESULTS

The results are reported in Table 1 and Table 2.

**Table 1:** Bone–implant mismatch (expressed as the percentage of extra implant thickness relative to the bone resection) in the different knee compartments according to coronal knee alignment.

	Varus HKA ≤ 177° (*N* = 50)	Neutral 177° < HKA > 183° (*N* = 24)	Valgus HKA ≥ 183° (*N* = 26)	Total (*N* = 100)	*p* value
%IMP medial tibia	64.0 ± 43.6	71.8 ± 57.4	55.2 ± 49.4	63.5 ± 48.3	0.581
	62.3 [37.6, 89.8]	59.5 [27.3, 89.8]	52.3 [23.1, 83.6]	59.5 [27.3, 89.8]	
%IMP lateral tibia	38.0 ± 45.8	35.1 ± 38.8	55.1 ± 65.3	41.8 ± 50.2	0.471
	20.9 [−2.2, 63.7]	20.9 [17.7, 48.7]	37.6 [11.1, 59.5]	20.9 [7.5, 59.5]	
%IMP medial posterior femur	−17.7 ± 10.8	−18.8 ± 10.7	−14.6 ± 11.4	−17.2 ± 10.9	0.573
	−18.5 [−24.6, −11.3]	−18.5 [−24.6, −11.3]	−11.3 [−24.6, −11.3]	−18.5 [−24.6, −11.3]	
%IMP lateral posterior femur	12.1 ± 18.1	14.7 ± 19.8	21.0 ± 24.1	15.1 ± 20.3	0.437
	7.9 [−2.6, 20.9]	20.9 [−2.6, 20.9]	20.9 [11.1, 20.9]	20.9 [−2.6, 20.9]	
%IMP medial distal femur	10.7 ± 22.5	−2.4 ± 20.5	19.0 ± 32.8	9.8 ± 26.0	**0.031** **0.028** *
	8.8 [−2.9, 20.1]	−7.6 [−20.1, 8.8]	8.8 [8.8, 23.8]	8.8 [−5.3, 23.8]	
%IMP lateral distal femur	15.1 ± 41.3	25.6 ± 45.2	40.7 ± 72.4	24.3 ± 52.4	0.444
	3.0 [−2.9, 23.8]	16.3 [5.9, 43.5]	3.0 [−12.4, 70.8]	8.8 [−5.3, 43.5]	
%IMP trochlea	−19.4 ± 17.9	−19.9 ± 16.8	−17.5 ± 28.5	−19.0 ± 20.7	0.999
	−20.3 [−31.2, −5.1]	−20.3 [−31.2, −5.1]	−20.3 [−37.5, −5.1]	−20.3 [−31.2, −5.1]	
%IMP patella	−16.7 ± 8.0	−5.4 ± 18.5	−1.4 ± 30.7	−8.7 ± 21.9	0.282
	−13.7 [−17.5, −13.7]	−13.7 [−13.7, −13.7]	−13.7 [−13.7, −3.3]	−13.7 [−13.7, −13.7]	
%IMP femoro‐patellar joint	−17.2 ± 16.7	−17.1 ± 17.5	−12.9 ± 24.3	−16.0 ± 19.0	0.723
	−20.3 [−31.2, −5.1]	−16.3 [−23.9, −5.1]	−10.6 [−28.5, −6.5]	−16.3 [−31.2, −5.1]	
%IMP total without femoro‐patellar joint	18.2 ± 15.0	17.4 ± 11.3	22.3 ± 12.0	19.1 ± 13.4	0.496
	13.0 [11.2, 23.5]	17.9 [9.0, 24.5]	21.6 [8.9, 31.4]	14.4 [9.6, 29.7]	
%IMP total	13.5 ± 12.8	13.2 ± 10.1	16.3 ± 11.0	14.2 ± 11.7	0.422
	10.0 [7.1, 15.5]	11.6 [7.1, 21.7]	19.5 [5.3, 23.7]	11.6 [6.9, 21.7]	

*Note*: Data are presented as mean ± standard deviation and median [first quartile, third quartile]. IMP% of extra implant thickness relative to the bone resection.

Abbreviations: HKA, hip‐knee‐ankle angle; IMP%, implanted percentage.

* *p* value refers to Neutral (−2.4 ± 20.5) vs. Valgus (9.8 ± 26.0).

**Table 2:** Comparison of bone–implant mismatch (expressed as the percentage of extra implant thickness relative to the bone resection) medial versus lateral in the different compartments of the tibio‐femoral joint.

Comparison	Medial	Lateral	*p* value
IMP% tibia	63.5 ± 48.3	41.8 ± 50.2	**0.0014**
	59.5 [27.3, 89.8]	20.9 [7.5, 59.5]	
IMP% posterior femur	−17.2 ± 10.9	15.1 ± 20.3	**<0.0001**
	−18.5 [−24.6, −11.3]	20.9 [−2.6, 20.9]	
IMP% distal femur	9.8 ± 26.0	24.3 ± 52.4	**0.016**
	8.8 [−5.3, 23.8]	8.8 [−5.3, 43.5]	

*Note*: Data are presented as mean ± standard deviation and median [first quartile, third quartile]. IMP% (implanted) percentage of extra implant thickness relative to the bone resection.

Among the 100 patients included in the study: 50 had a varus alignment (HKA ≤ 177°), 24 had a neutral alignment (177° < HKA < 183°) and 26 a valgus one (HKA ≥ 183°). The overall implant thickness was 14.2% greater than the bone resection.

**Figure 1:**
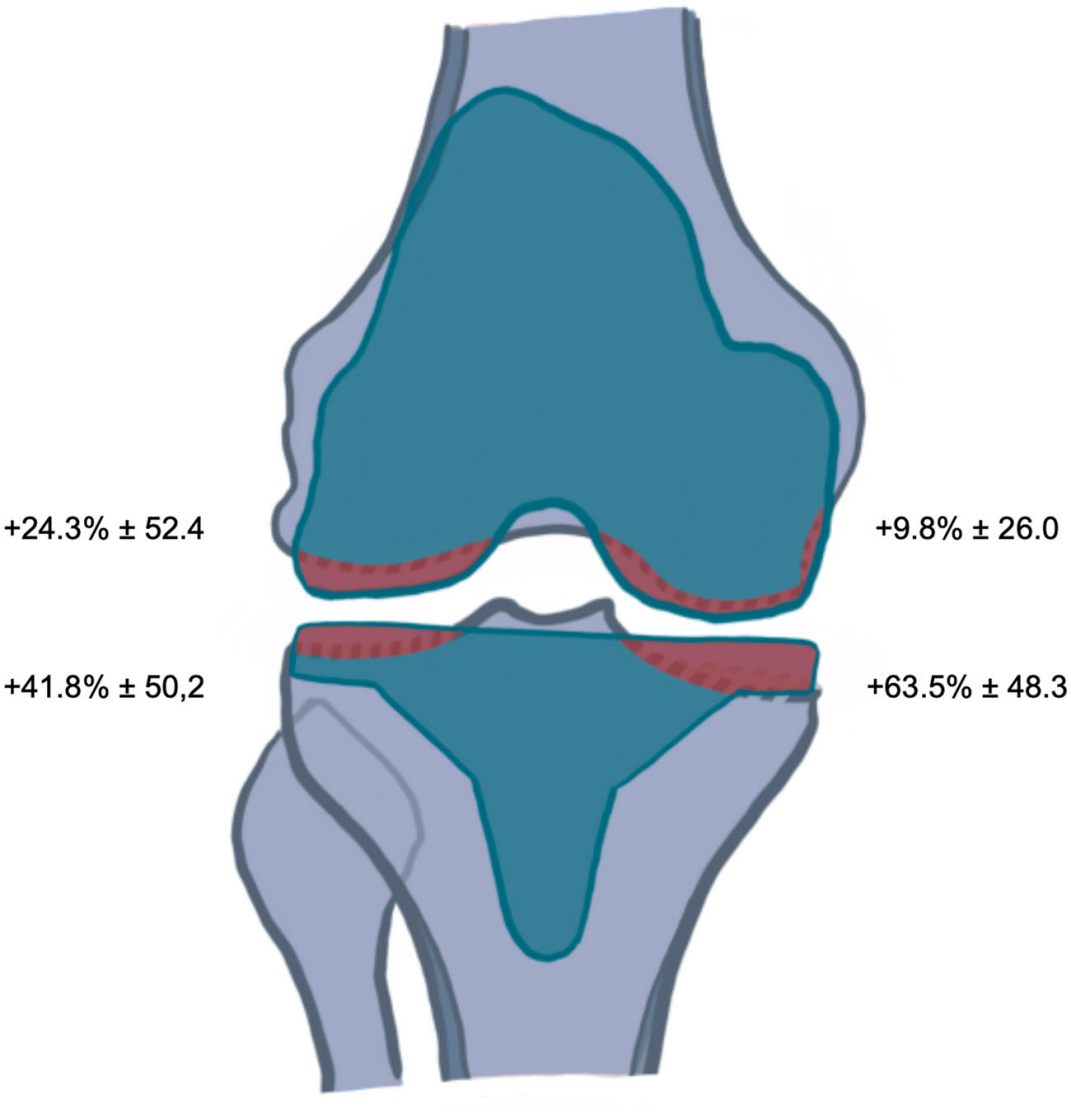
Bone implant mismatch in the tibia and in the distal femur. In red is represented a positive mismatch (more implant thickness) and in green a negative mismatch (more bone resection).

**Figure 2:**
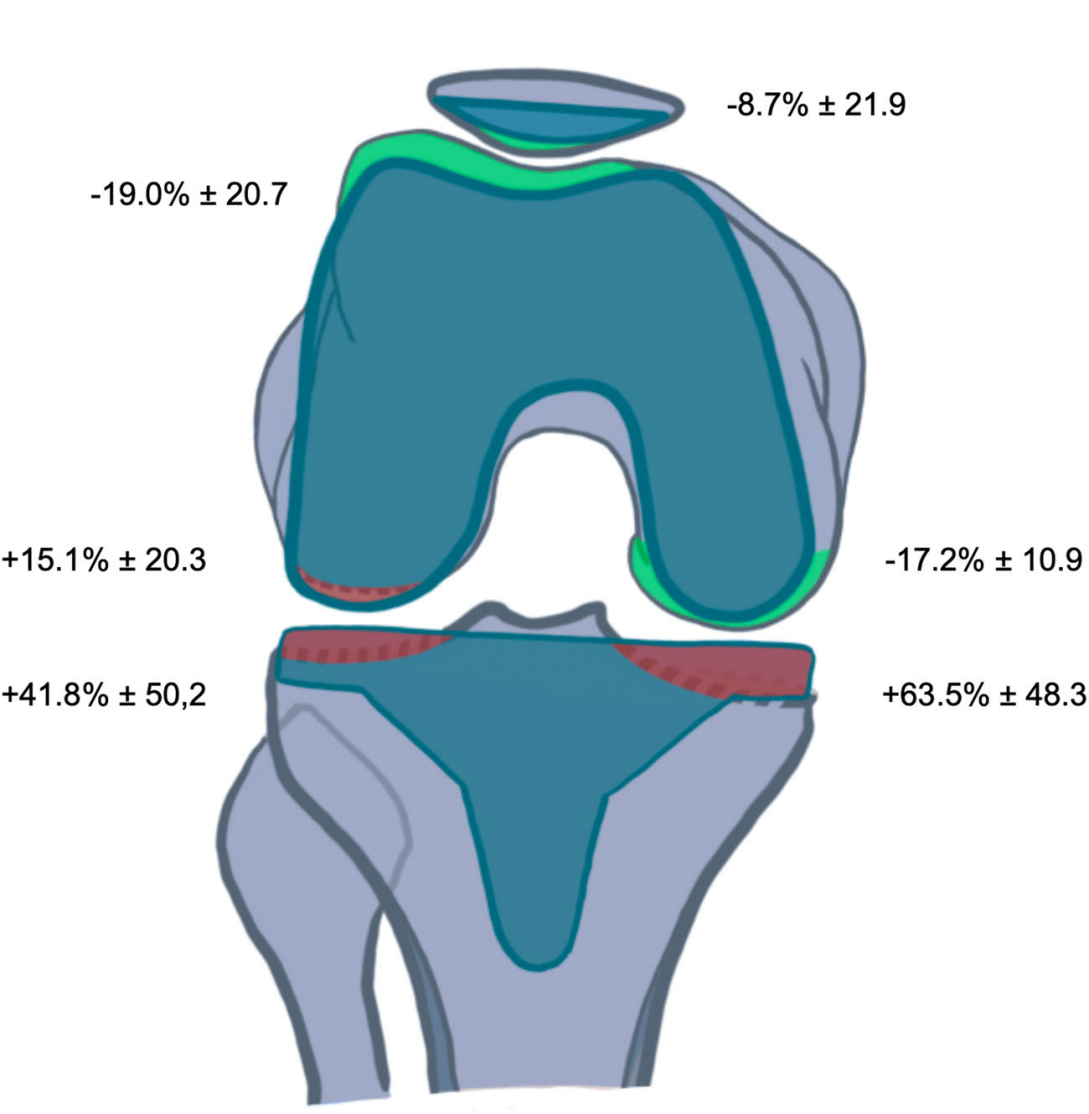
Bone implant mismatch in the tibia, posterior femur and in the patello‐femoral compartment. In red is represented a positive mismatch (more implant thickness) and in green a negative mismatch (more bone resection).

In the tibia, the implant thickness exceeded the bone resection by 63.5% ± 48.3 medially and by 41.8% ± 50.2 laterally, showing a significant statistical difference between medial and lateral (*p* = 0.0014). In the posterior femur, the implant thickness was 17.2% ± 10.9 less than the bone resection medially and 15.1% ± 20.3 more laterally (*p* < 0.0001). In the distal femur, the implant thickness exceeded the bone resection medially by 9.8% ± 26.0 and laterally by 24.3% ± 52.4 (*p* = 0.016). The implant thickness was 19.0% ± 20.7 less than the bone resection in the trochlea and 8.7% ± 21.9 less in the patella, resulting in 16.0% ± 19.0 less implant thickness in the overall femoro‐patellar compartment than the bone resection (Table 2). Figures 1 and 2 show the bone–implant mismatch in the various knee compartments.

The statical analysis did not show any significant statistical difference in bone–implant mismatch between the three alignment phenotypes, except in the medial distal femur, where the implant thickness was 2.4% ± 20.5 less than the bone resection in neutral knees while in valgus knees was 19.0% ± 32.8 more (*p* < 0.028) (Table 1).

## DISCUSSION

The main finding of this study is that the overall implant thickness, considering all compartments, is 14.2% greater than the bone resection, thus confirming our first hypothesis. Secondarily is also confirmed as there is bone–implant mismatch in each compartment of the knee with a significant difference medially compared to laterally in the tibio‐femoral joint. Finally, the consideration that the bone–implant mismatch in each compartment of the knee may differ significantly depending on the preoperative knee alignment phenotype, is refused, except considering the medial distal femur.

To the best of the author's knowledge, this is the first study to quantify and analyze the bone–implant mismatch in an OTS TKA with MA and to correlate these findings with the preoperative coronal knee alignment phenotype. As shown in the results section, there is a significant difference in bone–implant mismatch between the medial and lateral compartments in the distal femur, in the posterior femur and in the tibia. Specifically, the implant thickness exceeds the bone resection by 63.5% ± 48.3 in the medial tibia and by 41.8% ± 50.2 in the lateral tibia (*p* < 0.0001). In contrast, the femur showed an opposite pattern, with only 9.8% ± 26.0 extra implant thickness in the distal medial femur and 24.3% ± 52.4 extra implant thickness in the distal lateral femur (*p* < 0.0001). This distribution leads probably to an increase in both the medial proximal tibial angle (MPTA) and the lateral distal femoral angle (LDFA). Consequently, this may contribute to a modification of the JLO, which would tend to increase, shifting towards a neutral or proximal apex JLO phenotype, according to CPAK classification [19]. This pattern in JLO modification after TKA with MA is confirmed in literature. In their study Sappey‐Merinier et al. [25] showed that when performing TKA with MA, most of the patients had an increase in JLO, in their study they reported that preoperatively 63.5% of the patients had an apex distal JLO phenotype, 35.4% a neutral one and 1.1% an apex proximal phenotype; postoperatively only 8.5% still had an apex distal JLO while the majority, 81.9% had a neutral JLO and just a few (9.6%) an apex proximal. Cordan et al. [7] in their study also found a significant increased postop JLO in patients undergoing TKA with MA, as our study reported. Furthermore, Hirschmann et al. [26, 27] in their studies simulating bone resections using a model, demonstrated that both in varus and neutral knees phenotypes, MA tends to determine a proximalization of the joint line (JL) medially and a distalization laterally: this is in line with our results, confirming a pattern of increasing JLO (towards an apex proximal phenotype) after TKA with MA. It is also interesting to note that this pattern does not depend on the preoperative coronal alignment phenotype (varus, valgus and neutral) since, as shown in Table 1, there are no significant differences in bone–implant mismatch between the various alignments, except for a significant difference at the medial distal femur. The implant thickness in the medial distal femur was 2.4% ± 20.5 less than the bone resection in neutral knees while in valgus knees was 19.0% ± 32.8 more (*p* < 0.028), this means that in valgus knees there is an under‐resection of the distal medial femoral condyle compared to neutral ones. This may be attributed to the fact that valgus alignment often originates from femoral altered anatomy, typically characterised by metaphyseal deformity [31].

Considering the bone–implant mismatch in the posterior femur, the implant thickness is 17.2% ± 10.9 less than the bone resection on the medial side and 15.1% ± 20.3 more on the lateral side. According to the MA principles described by Insall [15], to achieve optimal gap balance in flexion, femoral posterior bone cuts are made in slight external rotation (using various reference parameters, such as the transepicondylar axis TEA, the posterior condylar axis PCA, or the anteroposterior AP axis). This leads to over‐resection of the medial posterior condyle and under‐resection of the lateral posterior condyle in order to achieve a balanced rectangular flexion gap. This is in accordance with our results, as 17.2% less posteromedial implant thickness and a 15.1% more posterolateral implant thickness determine a slight external rotation of the prosthetic component. Another interesting point is that in valgus knees this extra‐rotation is greater with 21.0% ± 24.1 more implant thickness (vs. 14.7% ± 19.8 in neutral knees and 12.1% ± 18.1 in varus knees) than bone resection in the lateral posterior femoral condyle. However, there was no statistically significant difference between the 3 values, probably due to a high standard deviation. The need of a greater femoral component external rotation in valgus knees to compensate the postero‐lateral cartilage worn or the lateral condyle hypoplasia, has been widely described in numerous studies in literature [18, 23].

Regarding the patellofemoral joint, our results clearly showed an important bone–implant mismatch in this compartment. The implant thickness is 19.0% ± 20.7 less than the bone resection in the trochlea and 8.7% ± 21.9 less in the patella, resulting in 16.0% ± 19.0 less implant thickness in the femoro‐patellar compartment than the bone resection. It is important to note that the patella was resurfaced in only 46 cases. This significant bone–implant mismatch is in our opinion due to different factors. First, most commercially available prostheses exhibit a degree of dysplasia in the trochlea. As reported by Saffarini et al. [24] in recent TKA designs trochlear compartments exceed radiologic signs of trochlear dysplasia by 2°–5°. Specifically, their study demonstrates that the sulcus angles remain 3°–6° greater (shallower) in prosthetic trochlear compartments. Secondly recent studies show that TKA performed with MA and using an off the shelf implant design does not restore the native trochlear groove [17, 20]. Reducing the implant thickness in PF compartment might compensate the dysplasia of the trochlea, lowering the risk of maltracking, instability, anterior pain and ROM limitation [16]. It should be noted that by resecting more bone in the trochlea compared to the implant thickness there is a risk of femoral notching [28, 30], which never occurred in our cohort of patients.

In none of the evaluated parameters (except at the distal femur) there was a significant difference in bone–implant mismatch according to knee alignment. This result suggests that, in achieving MA in TKA, the type of preoperative coronal alignment phenotype (varus, valgus or neutral) does not seem to determine our bone cuts. Instead, JLO seems to be a likely more determinant factor. Since JLO tends to decrease postoperatively moving toward a neutral value, probably the thickness of the bone cuts, at least in the coronal plane will be determined by its preoperative value.

The fact that in our study the overall implant thickness is 14.2% greater than the bone resection—and reaches 19.1% when excluding the patello‐femoral joint— likely represents in part a compensation for cartilage loss due to wear in osteoarthritic knees and meniscal removal during operation. This extra volume helps to restore the bone, cartilage and soft tissue loss. However, some of this extra implant thickness may be attributed to the type of alignment used. Kinematic alignment [13, 22] aims to preserve or restore the constitutional alignment, by aligning implants to the native condylar and tibial joint lines, and by matching component thickness of the femoral condyles and tibia to that of bone resection, plus a small additional amount to account for cartilage that has worn. This would lead probably to a smaller overall bone–implant mismatch and a smaller difference between medial and lateral bone–implant mismatch, minimally altering the natural knee spaces. However, these altered joint spaces are probably not due only to the alignment technique but also to the use of OTS prostheses. Such standardised implants may not perfectly conform to the patient's unique anatomy [1, 2]. By contrast, a custom‐made prosthesis would likely provide a more tailored fit to the patient's bone structure [3, 4]. It is likely that a custom‐made prosthesis combined with a personalised alignment [8] approach could offer the best solution to achieve optimal balance in joint spaces.

## LIMITATIONS

Our study has several limitations. First, it lacks postoperative long‐leg anteroposterior weightbearing radiographs, which would allow for an assessment of postoperative HKA. Additionally, parameters such as aHKA, MPTA and LDFA were not calculated. It does not account for cartilage wear, soft tissue thickness (including meniscal structures), or the ligamentous envelope, factors which could influence compartmental balancing together with the PE insert dimension. Moreover effect size measures were not reported.

## CONCLUSIONS

In mechanically aligned OTS TKA, implant thickness exceeds bone resection by an average of 14.2%, with a consistent bone–implant mismatch observed in all tibiofemoral compartments—significantly greater medially than laterally. Conversely, in the patellofemoral joint, bone resection surpasses implant thickness. Preoperative coronal alignment phenotype does not significantly influence this mismatch. These findings highlight a systematic discrepancy between bone resections and implant geometry, suggesting potential benefits from adjusted surgical techniques or implant design modifications to improve anatomical congruence and joint kinematics.

## AUTHOR CONTRIBUTIONS

All authors have given final approval of the submitted manuscript and their agreement to be accountable for all aspects of the work in ensuring that questions related to the accuracy or integrity of any part of the work are appropriately investigated and resolved. All authors have made substantial contributions to the design of the work and manuscript writing. All authors were involved in drafting the work or revising it critically for important intellectual content.

## CONFLICT OF INTEREST STATEMENT

The authors declare no conflicts of interest.

## ETHICS STATEMENT

The study was approved by the Institutional review board (IRB N° CTS‐P‐2024‐018‐FC). All patients gave valid consent to participate.
